# Design, Fabrication and Measurement of Full-Color Reflective Electrowetting Displays

**DOI:** 10.3390/mi13112034

**Published:** 2022-11-21

**Authors:** Guisong Yang, Benyou Wang, Zhiqiang Chang, Qing Liu, Linwei Liu

**Affiliations:** 1 College of Electronics and Information Engineering, West Anhui University, Lu’an 237012, China; 2 Guangdong Provincial Key Laboratory of Optical Information Materials and Technology and Institute of Electronic Paper Displays, South China Academy of Advanced Optoelectronics, South China Normal University, Guangzhou 510006, China

**Keywords:** electrowetting display, full color, control system, fabrication process

## Abstract

We designed, fabricated and measured full-color, reflective electrowetting displays (EWDs). The display system is composed of three-layer cyan, magenta and yellow EWD elements fabricated with standard photolithographic techniques. The EWDs were driven successfully by the proposed control system and the measurement results show that the electro-optical performance was improved. The aperture ratio of the EWD element can be tuned from 0 to ∼80% as the applied voltage is changed from 0 to 30 V. The response time and the color gamut were measured to be ∼18 ms and ∼58% NTSC, respectively. This paper makes it possible for large numbers of reflective full-color EWDs to be fabricated directly, with advantages of saving power significantly by 85% and no eye irritation compared with LED displays.

## 1. Introduction

Reflective displays such as electrowetting displays (EWDs) [1,2,3], electrophoretic displays (EPD) [4,5], and cholesteric LCDs [6,7] have been reported, which utilize ambient light to illuminate the screen, thereby providing superior energy efficiency, sunlight readability, and reading comfort. Among them, EWDs are becoming much more promising technology because of their low power consumption [2,8], high white state reflectance [9], fast switching [10], flexibility [11], multi-gray scale [12,13] and full color [14,15,16].

The one-layer [1,17] and three-layer [2,14] architecture full-color EWDs have been proposed in previous publications. The one-layer full-color EWD studies were mainly focused on dividing the display area into individual RGBK (red, green, blue and black) oil switches or using an RGB color filter with black oil. However, the resolution with individual RGBK oil switches approach is limited, since a full-color pixel has 4 × the area (RGBK) of a monochromatic pixel. In addition, individual RGBK color oils in 1 pixel can only be dosed by low-efficiency serial dosing techniques such as ink-jet-printing (IJT). Furthermore, using an RGB color filter with the black oil method will reduce the brightness of the display due to light adsorption. For previous three-layer architectures, the complicated fabrication process is the drawback. Additive RGB and subtractive cyan, magenta and yellow (CMY) color-mixing are the two main paths to generate colors for displays and are discussed in detail elsewhere [18]. For reflective displays, the RGB system relies on filtering down the incident light to red, green and blue primaries (effectively eliminating 2/3 of the incident light) and then using these filtered primaries in combination with grayscale to create a full-color image. If we choose the subtractive CMY system, there is no pre-mixing filtering, so the theoretical maximum of this type of color mixing is 100%, as is proven by color print on paper.

In this paper, we designed, fabricated and measured the reflective full-color EWD elements. The colors of the EWD elements are displayed by using a subtractive filtering technique that mixes the primary 3 CMY oil-filled layers. Three stacked layers are easily fabricated with the same photolithographic process, in which color, greyscale and intensity can be independently achieved. The electro-optical performance of the presented design is measured by the standard measurement setup, the results demonstrate its excellent optical control and efficient color mixing. More importantly, the presented system could provide proper illumination and color changing which has the dual benefits of energy-saving and no radiation in comparison with LED displays.

## 2. Materials and Methods

### 2.1. CMY Color Mixing Approach

**Figure 1:**
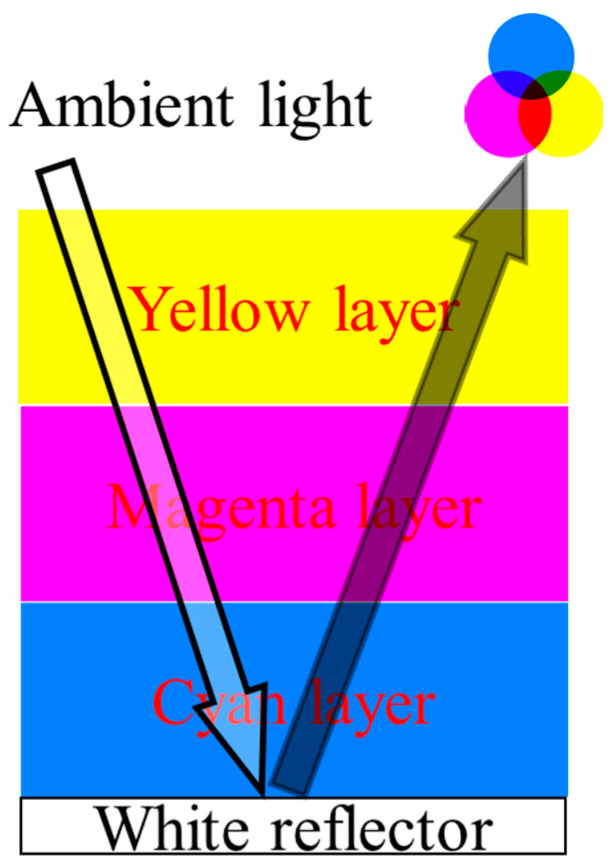
The cross-sectional structure of 3-layer subtractive color EWD.

The full-color EWD element panel consists of 3 separately contacted CMY layers of EWDs placed in a vertical stack and coupled by transparent UV-cured glue, in which color, greyscale and light intensity can be independently achieved. As mentioned above, the architecture provides the highest efficiency and resolution, as well as the simplest means for fabricating full-color EWD demonstrated to date. As shown in Figure 1, the color mixing paradigm is based on 3 colored layers of EWD stacked on a white reflector with all the benefits of reflection of ambient light, each layer filters out (absorbs) the wavelength of the light opposite to the color that is displayed, therefore a combination of 3-layer primary subtractive oil filters will result in black. The primary colors CMY can be displayed by turning layers “ON” and “OFF” and other colors (secondary, tertiary and further) can be displayed by varying the opening aperture of the oil in the layers, thus partly filtering the light. In this way, all colors can be produced in every pixel of the EWD element by varying the reflected light intensity in the layers.

### 2.2. ON/OFF Operating Principle of the EWD Pixel

**Figure 2:**
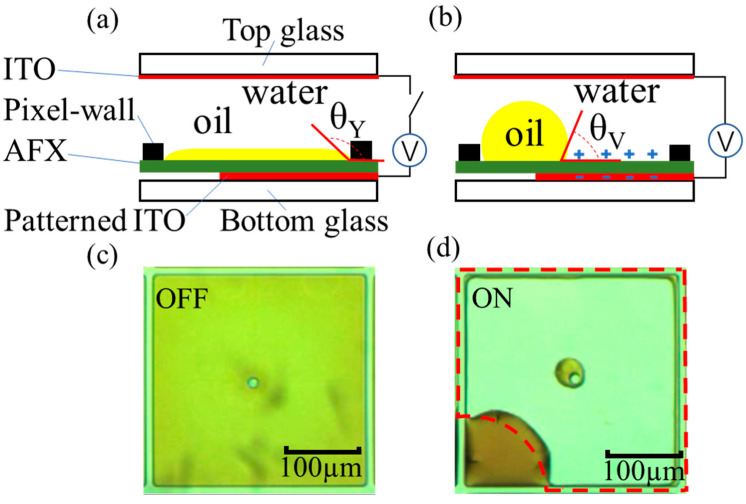
Operating principle of the EWD pixel. (**a**) Cross-sectional view of OFF state. (**b**) Cross-sectional view of ON state. (**c**). The microscope image of the pixel OFF state. (**d**) The microscope image of the pixel ON state.

As for the OFF and ON operating principle of the square pixel of each EWD layer, the cross-sectional schematic and the micrograph of the switching pixel were illustrated in Figure 2. The two-phase immiscible insulating oil and conducting water liquids completely covers the commonly used Teflon AF 1600X (AFX) for the hydrophobic surface of the pixel, exhibiting the color of the oil in the off-state without voltage, as shown in Figure 2a,c. When a voltage was applied between the top indium tin oxide (ITO) and the bottom notched pattern ITO electrode, as shown in Figure 2b,d, the initial contact angle $\theta_{Y}\;$ of the conducting water on AFX will be reduced to $\theta_{V}\;$ according to the Young–Lippmann equation,

(1) $$\cos\theta_{V}=\cos\theta_{Y}+\frac{CV^{2}}{2\gamma_{AO}}$$

where C is the capacitance per unit area of the hydrophobic and dielectric (F/m^2^), V is the DC voltage and $\gamma_{AO}$ is the interfacial surface tension between the water and oil phases (N/m). The oil film is pushed aside to the corner by the shape deformation of the water, showing the voltage-on state of the transparent pixel.

### 2.3. Driving Control System of the EWDs

**Figure 3:**
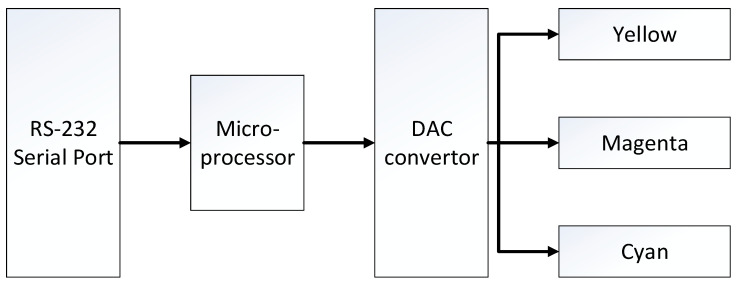
Schematic diagram of the full-color EWDs control system.

Similar to most LED display control systems [19,20,21], the schematic of the EWDs control system in this paper is shown in Figure 3. The main components of the system are: microprocessor controller (ATmega16, ATMEL, San Jose, CA, USA), RS-232 serial port (MAX3232, TI, Dallas, TX, USA), digital analog convertor (LTC2642CMS, LINEAR, Milpitas, CA, USA) and 3 CMY EWD layers. The microprocessor receives the brightness, color and grayscale data instructions sent from the host computer through the RS-232 serial port, converts the signal into a digital PWM voltage waveform, and sends the waveform signal to the DAC converter unit. Finally, 3-way different analog voltages (0~30 V) were generated for the three separated CMY EWD display layers to change the color and the brightness. Through the above components, the system will eventually output the required control waveform for the EWD cells. It should be noted that there was a “backflow” phenomenon during the driving process of the EWD [22,23,24]. When the voltage is continuously applied to the two electrodes of the EWD pixel, the oil will shrink to the corner and the pixel will open. However, after a few seconds, the oil will gradually spread to the entire pixel surface, and the pixel close again. In order to overcome this phenomenon, it is necessary to apply a reset waveform (0 V) in the control waveforms periodically.

### 2.4. Fabrication Process of the EWDs

**Figure 4:**
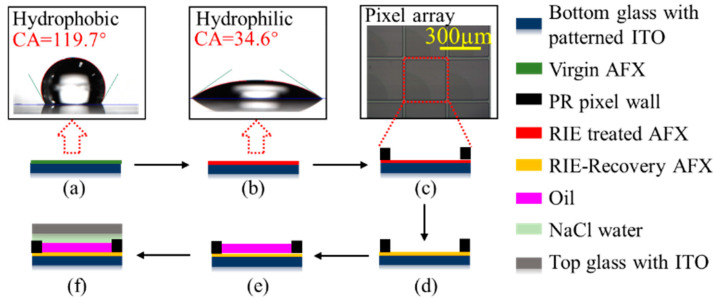
The fabrication process of the 1-layer EWD. (**a**) Coat AFX. (**b**) RIE activation. (**c**) Pixel wall structuring. (**d**) Reflow. (**e**) Oil filling. (**f**) Sealing.

To demonstrate a full-color EWD element, 3 colored layers are fabricated by the same process except for the color of the oil, performed as in Figure 4.

First, the bottom glass with notch pattern ITO arrays (Jimmy Glass Technology Ltd., Foshan, China) was cleaned in an LCD cleaning line prior to use. The AFX solution (Dupont, Shanghai, China) was coated on an ITO-glass surface by a screen printer with a thickness in the range of 600 nm–800 nm, the substrate was cured on a hot plate at 85 °C for 5 min and then in an oven at 185 °C for 30 min. Thus, a virgin hydrophobic AFX film was obtained and the measured contact angle (CA) of the NaCl water on the virgin AFX is about 119.7°. In addition, the virgin AFX surface was treated by a reactive ion etching (RIE) process, tuning the hydrophobic AFX surface to become a hydrophilic RIE-treated AFX film, which facilitates its subsequent coating by hydrophilic photoresist (PR). The measured CA of the NaCl water on the RIE-treated AFX was 34.6°. Afterward, the PR (SUNTIFIC Company, Weifang, China) was coated onto the RIE-treated AFX surface via slit coating. The thickness of the PR was uniform (+/−2%) in the range of 5–8 μm. A conventional lithography process was implemented to fabricate the PR film into pixel wall arrays in an EWD device. Next, a thermal reflow process with a temperature of ~200 °C for 2 h was used to return the surface of the hydrophilic RIE-treated AFX back to its native hydrophobic RIE-recovery AFX. Then, the oil was filled by self-assembly technology using of its surface tension. The substrate as manufactured above is slowly lowered through an oil film floating on NaCl water, using filling equipment developed in-house. Finally, using a conventional screen printer, a UV-curable sealing agent was printed on the top ITO glass, sealed with the above oil-filled substrate glass using external pressure. The detailed fabrication parameters of full-color EWD are shown in Table 1.

Three CMY-separated layers were stacked together by the dedicated machine. UV-cured glue was used to integrate them. Precise pixel alignment under a microscope should be performed to achieve excellent electro-optic performance.

### 2.5. Electro-Optical Performance Measurement Setup

**Figure 5:**
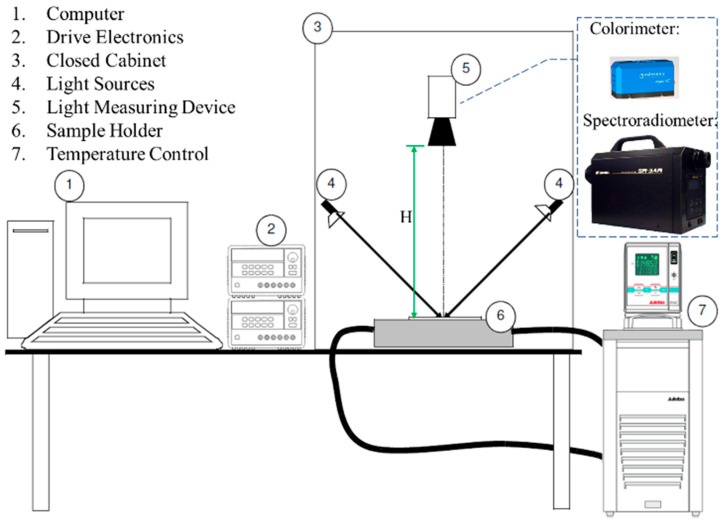
Layout diagram of EWD electro-optical measurement setup.

The electro-optical performance of the full-color EWD element shall be measured under specific and well-defined conditions of illumination and detection in order to be reproducible since it reflects the ambient light to illuminate the screen. The electro-optical measurement setup is shown in Figure 5. A Windows-based computer controls the temperature in the closed cabinet to be standard 25 °C ± 3 °C with a temperature control unit, utilizing the drive electronics to generate proper power supply and electrical waveform connecting to the EWD under test on the sample holder. The optical setup can be shielded from ambient light by closing the cabinet, then the EWD is illuminated only by two Philips brilliant line dichroic 12 V/20 W halogen lamps as 45° incident light sources in the cabinet. The light measuring device (LMD) is positioned in a 45°/0° geometry and kept at a height (H) above the EWD element to ensure only diffuse reflection of the sample display is measured.

## 3. Results

### 3.1. The Aperture Ratio and the Driving Voltage

**Figure 6:**
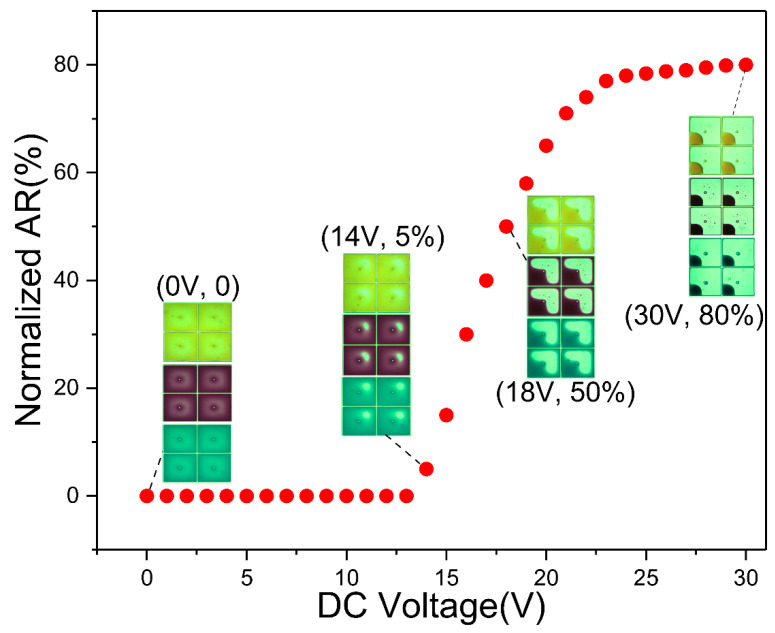
Normalized AR versus the applied voltage of the EWD elements.

The aperture ratio (AR) is used to measure the white area ratio (i.e., substrate percentage) in a pixel. It is defined as,

(2) $$\mathrm{AR}\%=\left(1-\frac{A_{oil}\left(V\right)}{A_{pix}}\right)\times 100\%\;$$

where $\;A_{oil}$ and $\;A_{pix}$ denote the area occupied by the oil and the overall area of the pixel, respectively, and *V* is the voltage applied to the device. To study the AR change of the EWD element, an optical microscope was used to record cyan, magenta and yellow layers oil movement in pixels. The AR was extracted from the microscope video snapshots using a MATLAB routine, as shown in Figure 6, and the insets show the microscope image of four adjacent pixels of each layer during 0–30 V actuation. At *V* = 0 V, three layers of oil homogeneously covered the entire pixel and the AR is 0. At *V* = 14 V, three layers of oil film began to rupture, the ~5% AR was achieved and the AR increased gradually when the applied voltage exceeded the threshold voltage 14 V. At *V* = 18 V, ~50% AR was achieved and half the area of the pixel was occupied by the colored oil. The largest AR was measured to be ∼80% at *V* = 30 V, in which the oil was driven to the pixel corner. After removing the voltage, the oil droplet can return to its original state quickly. The AR compared well with the previous publication (AR = 67%, *V* = 30 V) [25].

### 3.2. The Response Time

**Figure 7:**
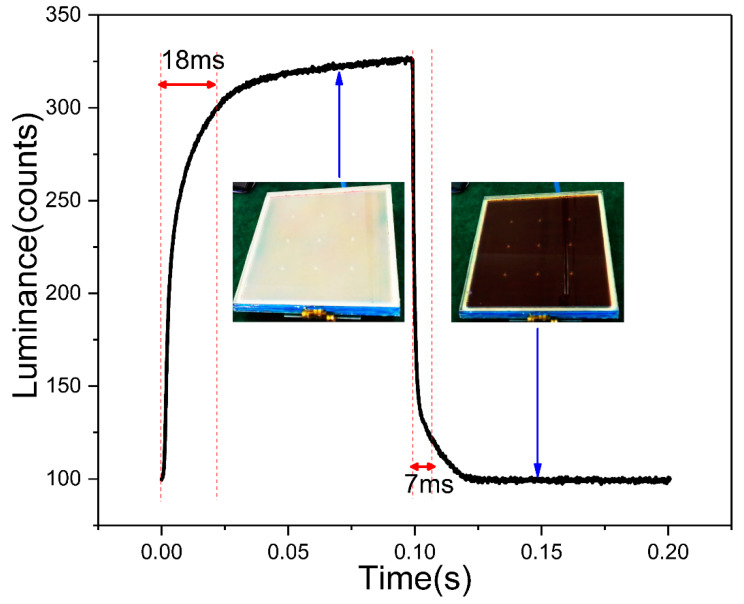
The optical response measured by colorimeter of the EWDs.

As an optical display element, the response time is an important parameter. Figure 7 shows the optical response of full-color EWD element measured in the optical setup. Figure 4 shows where the optical colorimeter (Arges 45, Admesy, Ittervoort, The Netherlands) was used as the LMD and 30 V DC voltage was applied at the duty cycle of 50% 5 HZ frequency as a rectangular electrical waveform was generated by drive electronics. When the EWD element was switched to the “ON” state, higher luminance was measured, and vice versa when the device was switched “OFF”, as shown in the insets of Figure 6. We define 90% of the maximum luminance change as the effective display, the switching on time is 18 ms and the switching off time is 7 ms, in the video range. The response time compared well with previous work of the EWD (32 ms) [26]. The highest luminance was ~330 units and the lowest was ~100 units, which means the highest contrast ratio that could be reached was ~3.3.

### 3.3. The Color Gamut

**Figure 8:**
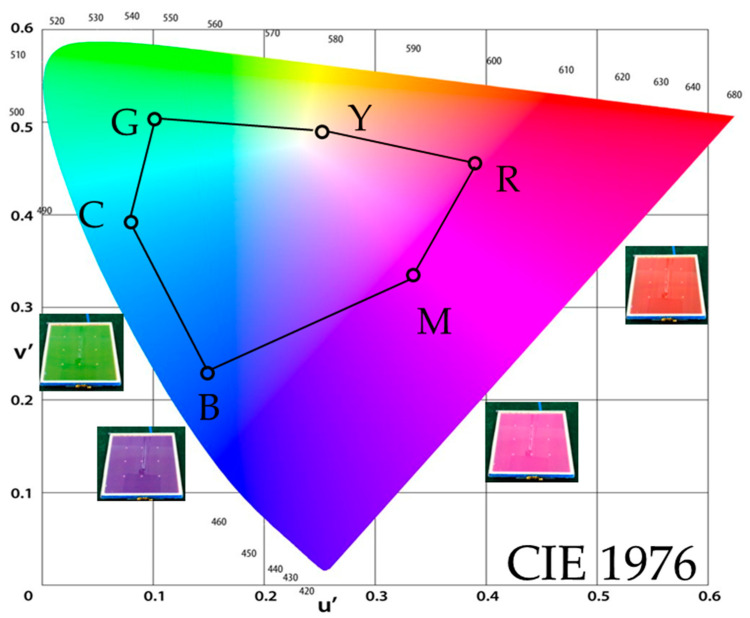
Color gamut in CIE 1976 measured by a spectroradiometer. The insets show pictures of red, green, magenta and cyan EWDs, respectively.

The color gamut is another important parameter of the optical display element. To evaluate the color gamut in the electro-optical measurement setup as shown in Figure 5, the spectroradiometer (SR-3AR, Topcon, Tokyo, Japan) was used as an LMD positioned normal to the EWD surface (H = 20 cm) and the colorimetric values were obtained through a numerical conversion of the measured spectral radiance data. As a result, six reference colors were specified successfully in CIE 1976 (u’, v’) color space and 58% NTSC color gamut was achieved, as shown in Figure 8. The color gamut is comparable to the previous report of the author (63% NTSC) [22]. The insets show colored display pictures of the EWDs.

The CMY color mixture method shows, as was expected theoretically, the typical gamut of a printer. Some deviation in the blue coordinate from (printer) standards is apparent. However, some strategies (e.g., the optimization of the oil concentration and thinner substrate) could improve the color gamut of the EWD further.

Table 2 lists a comparison of EWD proposed in this paper with the traditional LED display system, which shows its dual benefits: reducing energy by ~85% and improving viewing comfort.

## 4. Conclusions

In conclusion, we have demonstrated a reflective display system that combined three-layer CMY-stacked full-color EWD elements. Three layers of the EWD element were fabricated by the same photolithographic techniques except for the color of the oil. In addition, the electro-optical performance was obtained in the standard measurement setup, the results showed that the EWD element exhibits from 0 to ∼80% aperture ratio varying with the applied voltage from 0 to 30 V, the reasonable response time and color gamut in CIE 1976 color space obtained were ∼18 ms and ∼58% NTSC, respectively. The reflective full-color EWD elements have a great potential in video-speed outdoor display applications, which offers the advantages of great energy efficiency and viewing comfort compared with LED displays.

## Figures and Tables

**Table 1:** Fabrication parameters of full-color EWD.

Panel Size (mm)	Pixel Size (μm)	PR (μm)	Pixel Wall Width (μm)	ITO (nm)	AFX (nm)
96 × 96	300 × 300	6.8	10	25	800

**Table 2:** Comparison of full-color EWD with LED display.

Display Technology	Power Consumption (mW/inch^2^)	Viewing Comfort	Display Mode
EWD	25	Good	Reflective
LED	170	Poor (radiation)	Emissive

## Data Availability

Not applicable.

## References

[1] Hayes R.A., Feenstra B.J. (2003). Video-speed electronic paper based on electrowetting. Nature.

[2] Heikenfeld J., Zhou K., Kreit E., Raj B., Yang S., Sun B., Milarcik A., Clapp L., Schwartz R. (2009). Electrofluidic displays using Young–Laplace transposition of brilliant pigment dispersions. Nat. Photonics.

[3] Shui L., Hayes R.A., Jin M., Zhang X., Bai P., van den Berg A., Zhou G. (2014). Microfluidics for electronic paper-like displays. Lab A Chip.

[4] Comiskey B., Albert J.D., Yoshizawa H., Jacobson J. (1998). An electrophoretic ink for all-printed reflective electronic displays. Nature.

[5] Kao W.C., Ye J.A., Chu M.I., Su C.y. (2009). Image quality improvement for electrophoretic displays by combining contrast enhancement and halftoning techniques. IEEE Trans. Consum. Electron..

[6] Kim K.-H., Jin H.-J., Song D.H., Cheong B.-H., Choi H.-Y., Shin S.T., Kim J.C., Yoon T.-H. (2010). Switching of liquid-crystal devices between reflective and transmissive modes using long-pitch cholesteric liquid crystals. Opt. Lett..

[7] Lu S.-Y., Chien L.-C. (2007). A polymer-stabilized single-layer color cholesteric liquid crystal display with anisotropic reflection. Appl. Phys. Lett..

[8] Giraldo A., Massard R., Mans J., Derckx E., Aubert J., Mennen J. (2011). 10.3: Ultra low-power Electrowetting-based Displays Using Dynamic Frame Rate Driving. SID Symp. Dig. Tech. Pap..

[9] Heikenfeld J., Smith N., Dhindsa M., Zhou K., Kilaru M., Hou L., Zhang J., Kreit E., Raj B. (2009). Recent progress in arrayed electrowetting optics. Opt. Photonics News.

[10] Lee P.T., Chiu C.-W., Lee T.-M., Chang T.-Y., Wu M.-T., Cheng W.-Y., Kuo S.-W., Lin J.-J. (2013). First fabrication of electrowetting display by using pigment-in-oil driving pixels. ACS Appl. Mater. Interfaces.

[11] Kim D.Y., Steckl A.J. (2010). Electrowetting on Paper for Electronic Paper Display. ACS Appl. Mater. Interfaces.

[12] Yi Z., Shui L., Wang L., Jin M., Hayes R.A., Zhou G. (2015). A novel driver for active matrix electrowetting displays. Displays.

[13] Liu L., Bai P., Yi Z., Zhou G. (2021). A Separated Reset Waveform Design for Suppressing Oil Backflow in Active Matrix Electrowetting Displays. Micromachines.

[14] You H., Steckl A. (2010). Three-color electrowetting display device for electronic paper. Appl. Phys. Lett..

[15] Guo Y., Zhuang L., Feng H., Zhong B., Henzen A., Groenewold J., Liu F., Deng Y., Tang B., Zhou G. (2021). Programmable Control of Two-Phase Fluid Interface Relative Motion in Electrowetting Device. Adv. Mater. Interfaces.

[16] Henzen A., Zhou G., Guo Y., Dou Y., Jiang H., Yang G., Tang B. (2019). 36-4: Full Color Active Matrix Video E-Paper. SID Symp. Dig. Tech. Pap..

[17] Ku Y.S., Kuo S.W., Huang Y.S., Chen C.Y., Lo K.L., Cheng W.Y., Shiu J.W. (2011). Single-layered multi-color electrowetting display by using ink-jet-printing technology and fluid-motion prediction with simulation. J. Soc. Inf. Disp..

[18] Simonot L., Hébert M. (2014). Between additive and subtractive color mixings: Intermediate mixing models. J. Opt. Soc. Am. A.

[19] Ng S.K., Loo K.H., Lai Y.M., Tse C.K. (2014). Color Control System for RGB LED With Application to Light Sources Suffering From Prolonged Aging. IEEE Trans. Ind. Electron..

[20] Visconti P., Lay-Ekuakille A., Primiceri P., Ciccarese G., Fazio R.d. (2017). Hardware Design and Software Development for a White LED-Based Experimental Spectrophotometer Managed by a PIC-Based Control System. IEEE Sens. J..

[21] Song Y., Feng Y., Ma J., Zhang X. (2011). Design of LED Display Control System Based on AT89C52 Single Chip Microcomputer. J. Comput..

[22] Yang G., Tang B., Yuan D., Henzen A., Zhou G. (2019). Scalable Fabrication and Testing Processes for Three-Layer Multi-Color Segmented Electrowetting Display. Micromachines.

[23] Lu Y., Tang B., Yang G., Guo Y., Liu L., Henzen A. (2021). Progress in Advanced Properties of Electrowetting Displays. Micromachines.

[24] Yang G., Liu L., Zheng Z., Henzen A., Xi K., Bai P., Zhou G. (2020). A portable driving system for high-resolution active matrix electrowetting display based on FPGA. J. Soc. Inf. Disp..

[25] Luo Z., Fan J., Xu J., Zhou G., Liu S. (2020). A novel driving scheme for oil-splitting suppression in Electrowetting display. Opt. Rev..

[26] Zeng W., Yi Z., Zhao Y., Zeng W., Ma S., Zhou X., Feng H., Liu L., Shui L., Zhang C. (2021). Design of Driving Waveform Based on Overdriving Voltage for Shortening Response Time in Electrowetting Displays. Front. Phys..

